# Supplementary material for: Enhancement of loudness discrimination acuity for self-generated sound is independent of musical experience
Source: PLoS One. 2021 Dec 7;16(12):e0260859. doi: 10.1371/journal.pone.0260859 (PMC8651135; doi:10.1371/journal.pone.0260859)
Supplement: Attachment — Submitted filename: Response to Reviewers.docx [file pone.0260859.s002.docx]

**Response to Reviewer #2:**

The revision clarified the preciously raised concerns. I appreciate the author’s efforts and recommend publication. There is just one minor comment about the added correlation analyses. It was not described clearly how exactly the author performed the analyses. For example, did they perform Pearson or Spearman correlation, or others? Did they perform the t-test on the correlations (l. 210-214)?

*We carried out Pearson’s product-moment correlation (Pearson’s correlation) and t-test on the correlations. We added this point in the method (l.170, 175) and the result (l.207). Thanks.*
